# Correlating symptoms to infectivity among vaccinated healthcare workers with COVID-19

**DOI:** 10.1017/ash.2023.344

**Published:** 2023-09-29

**Authors:** Abdulaziz Almulhim, Francine Touzard Romo, Leonard Mermel, Amy Mathers, Joshua Eby

## Abstract

**Background:** Directing COVID-19 diagnostic testing to healthcare workers (HCWs) who are likely to be infected has potential to reduce staffing shortages and decrease opportunity for in-hospital transmission; however, HCWs with COVID-19 may exhibit a range of symptoms. We assessed the burden of symptoms in relation to cycle threshold (Ct) values as a surrogate for viral shedding in vaccinated healthcare workers. **Methods:** We retrospectively reviewed employee health records of COVID-19–vaccinated employees who tested positive for SARS-CoV-2 between December 2020 and January 2022 at 2 academic hospital systems. We reviewed demographic data, reasons for testing including symptoms, exposure history, medical history, vaccination dates, Ct values, and genotypes when available. We compared mean Ct values between symptomatic and minimally symptomatic cases using independent sample *t* tests. Patients were defined as minimally symptomatic if they had no symptoms or a single symptom that is not cough, fever, or anosmia at the time of testing. Patients were defined as more symptomatic if they reported >1 symptom or cough, fever, or anosmia. **Results:** In total, 298 HCWs tested positive for COVID-19. Most positive cases were female (73%), white (78%), and had patient-facing roles (77%). Genotypic testing (n = 109) revealed that most genotypes belonged to the SARS-CoV-2 delta variant (AY lineages, B1.617.2). More cases were minimally symptomatic (62%) than were more symptomatic (38%). None required hospitalization during the study period. Mean Ct values (n = 141) showed no significant difference between more symptomatic and minimally symptomatic cases (19.8 vs 20.6; *P =* .40) (Fig. 1). Also, there was no significant difference in mean Ct value, comparing those with vaccination 90 days prior to positive (20.52 vs 19.88; *P =* .537). **Conclusions:** Our study shows no significant difference in cycle threshold values between minimally symptomatic and more symptomatic infections in vaccinated HCWs. In addition, HCWs exhibit high viral load even when infected within 90 days after vaccination. When considering whether to attend work, HCWs should be aware that mild symptoms and recent vaccination do not necessarily reflect low transmissibility and that they should follow CDC guidance regarding when to return to work.

**Figure:**
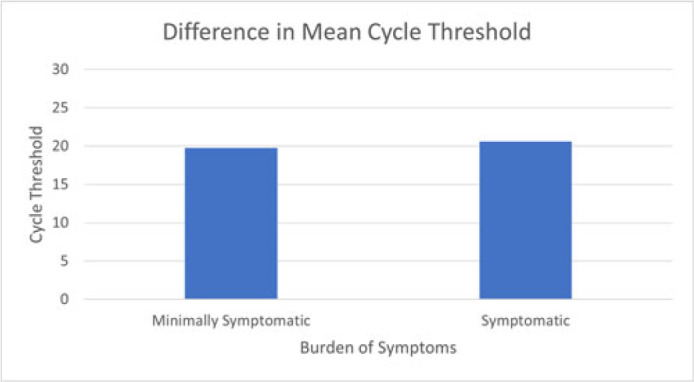

**Disclosures:** None

